# A randomized controlled study of exposure therapy as aftercare for alcohol use disorder: study protocol

**DOI:** 10.1186/s12888-016-0795-8

**Published:** 2016-04-21

**Authors:** Angelina Isabella Mellentin, Bent Nielsen, Anette Søgaard Nielsen, Fei Yu, Elsebeth Stenager

**Affiliations:** Unit of Psychiatric Research, Institute of Regional Health Services Research, University of Southern Denmark, Aabenraa, Denmark; Unit of Clinical Alcohol Research, Department of Clinical Research, University of Southern Denmark, Odense, Denmark; Innovation and Business Research Unit, Mads Clausen Institutet, University of Southern Denmark, Sønderborg, Denmark; Unit of Psychiatric Research, University of Southern Denmark, Sdr. Boulevard 29, 5000 Odense, Denmark

**Keywords:** Aftercare, Alcohol use disorder, Cognitive behavioural treatment, Cue exposure treatment, Smartphone application

## Abstract

**Background:**

It is well documented that individuals with Alcohol Use Disorder (AUD) respond well during evidence-based psychological treatment, but also that a large proportion relapses when discharged from treatment and confronted with alcohol in real life. Cue Exposure Treatment (CET) focuses on exposing individuals to alcohol cues in order to reduce cravings as well as the likelihood of relapse.

The aims of the study are: 1) to investigate whether CET aftercare delivered via a smartphone or in group sessions increases the effect of Cognitive Behavioural Treatment in groups of alcohol dependent individuals; 2) to investigate whether CET as a smartphone application is as or more effective than CET group therapy, and 3) to investigate whether CET as a smartphone application is more cost-effective than CET group aftercare and Aftercare as Usual.

**Design and methods:**

The study will be implemented as an investigator-blinded randomized controlled trial. A total of 300 consecutively enrolled alcohol use disorder individuals recruited from an alcohol outpatient clinic will be randomized into one of the three following aftercare groups after concluding primary treatment: (1) CET as a smartphone application; (2) CET as group therapy, and (3) Aftercare as Usual. It is hypothesized that the two experimental groups will achieve better treatment outcomes compared to the control group (3).

**Discussion:**

Individuals in the CET groups are given the opportunity to practise coping strategies during exposure to alcohol stimuli before being unavoidably confronted with alcohol and associated stimuli in real life. Thus, CET may help prevent patients from relapsing after concluding treatment, and in the long term. Moreover, the CET application has the potential to improve AUD treatment and continuing care by offering psychological treatment whenever and wherever the patient finds it convenient.

**Trial registration:**

ClinicalTrials.gov ID: NCT02298751

Registration date: 6 November 2014

## Background

Alcohol Use Disorder (AUD) is a widespread problem in Denmark*,* and it is a well-known fact that AUD leads to a substantial number of contacts with the treatment system, which constitutes a substantial burden on the healthcare system [1]. Individuals with AUD respond relatively well during evidence-based psychological treatment, but a large number of patients relapse when discharged from treatment and confronted with alcohol in real life [2, 3]. During evidence-based psychological alcohol treatment, in particular when applying Cognitive Behavioural Therapy (CBT), patients learn methods to help them identify high-risk situations for relapse. They also learn coping strategies for avoiding or confronting alcohol and associated high-risk situations [4–8]. However, patients are supposed to learn this by talking about what to do, not by actually being exposed to alcohol and practicing the strategies in vivo, as CBT is delivered when specifically targeting AUD. For the patient, the real challenge is to make use of coping strategies in real life outside the treatment setting; to actually use the strategies before or in the situation in question. All too often, the patient relapses when exposed to alcohol and associated stimuli and can afterwards only analyse and describe what went wrong. In other words, the challenge is to prepare the patient for his confrontation with alcohol, given that avoiding alcohol-related situations in a Danish context is almost impossible, and to provide the patient with strategies that can be used before he/she starts to drink, in order to prevent a relapse.

A reasonable means to achieve this goal is to teach the patients how to apply coping strategies to regain control over their alcohol cravings in their daily confrontations with alcohol and associated stimuli before the end of their contact with the treatment institution. Cue Exposure Therapy (CET) is a behavioural psychological approach that focuses on confronting alcohol cues in order to reduce cravings as well as the likelihood of relapsing. During CET, individuals are exposed to alcohol-related stimuli whilst their usual drink responses are prevented. Thus, by applying CET during the treatment course, patients are given an opportunity to practise coping strategies when exposed to alcohol in vivo before they are discharged from the treatment system. It is hypothesized that in this way the individual’s conditioned automatic responses will gradually extinguish over time, and that their cognitive control over cue reactivity will strengthen.

Because psychological treatment such as CET is a substantial socio-economic burden when delivered in individual sessions, the trend is towards delivering treatment through group sessions. Mental healthcare applications such as smartphone apps may have even greater potential, both in terms of cost-reduction in the healthcare system and by increasing the availability of evidence-based treatment [9]. However, whilst group sessions have been documented as effective (e.g. [6, 10]), behavioural healthcare apps targeting AUD need further exploration, despite promising preliminary results [11, 12].

### Aims

The aims of the study are:

1. To investigate whether manual-based CET delivered via a smartphone or in group sessions increases the effect of outpatient CBT treatment in AUD individuals
2. To investigate whether CET as a smartphone application is as effective as CET group aftercare and Aftercare as Usual
3. To investigate whether CET as a smartphone application is more cost-effective than CET group aftercare and Aftercare as Usual

## Design and methods

**Fig. 1:**
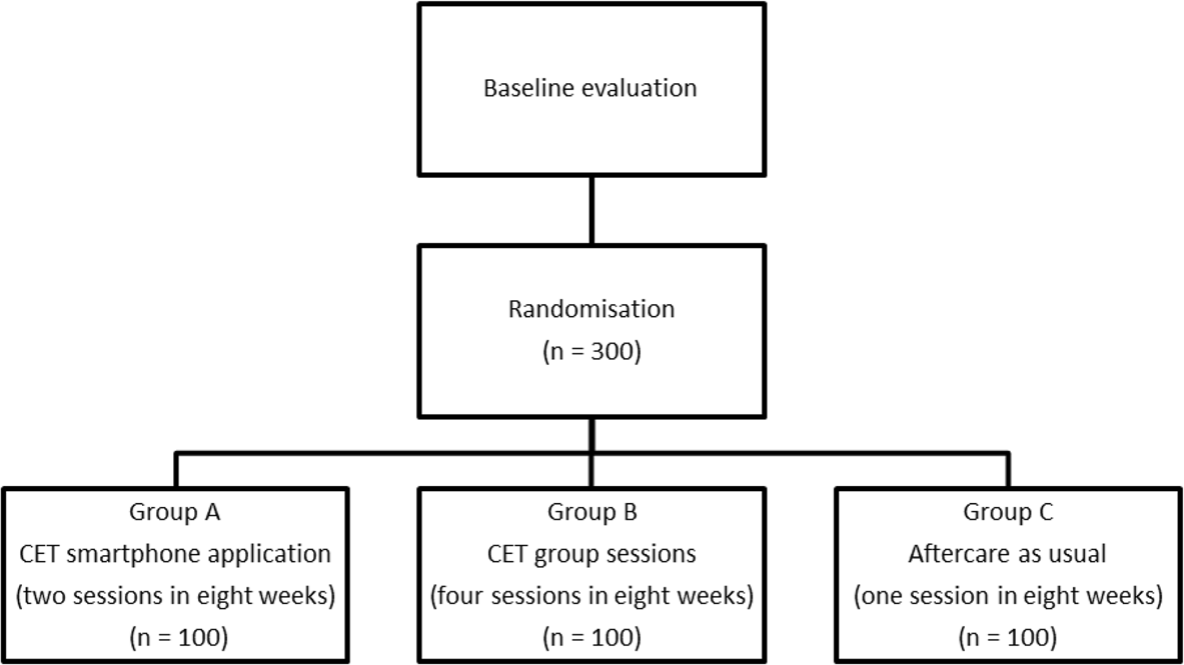
Flowchart

The study will be implemented as a parallel investigator-blinded randomized controlled trial. Randomisation occurs by means of computerized urn randomization. The random allocation sequence is generated by a statistician who has no contact with either patients or research assistants. As illustrated below in Fig. 1: Flowchart, a total of 300 consecutively enrolled AUD individuals will be recruited from an alcohol outpatient clinic after concluding a 3-month standard outpatient treatment course. The standard outpatient treatment course typically consists of eight sessions, and is based on cognitive behavioural therapy. The treatment course is planned together with the patient, and the therapy typically consists of psycho-education, functional analysis of drinking situations, development of coping strategies (e.g. waiting out till the urge passes, thinking about the negative consequences of drinking, thinking about positive consequences of sobriety, and intake of alternative food and beverage), problem-solving and homework between sessions.

In the present study the patient will be briefly informed about the aftercare project in the second-last session of the standard 3-month outpatient treatment course, and asked if he/she is willing to meet with a research assistant who will provide further information. If so, the patient is informed by the research assistant about this study’s aftercare project right after treatment conclusion. After informed consent, the baseline interview will be carried out, and patients fulfilling the inclusion/exclusion criteria will be randomized into one of the three aftercare treatment groups mentioned below by the means of a computer program. The inclusion criteria are as follows: patients must 1) accept participation in the study and sign written informed consent; 2) be aged between 18 and 60 years, and 3) have completed primary treatment. The exclusion criteria are: 4) patients who are not Danish speakers; 5) patients with acute psychotic disorders or severe cognitive impairment, and 6) patients with terminal somatic illness. Patients fulfilling the above criteria will be randomized into one of the three following aftercare treatment groups: (A) CET as a smartphone application (*n* = 100); (B) CET as group therapy (*n* = 100), and (C) Aftercare as Usual (*n* = 100). Patients in group (A) are encouraged to use the smartphone application four times a week for 8 weeks (a maximum of 36 sessions of approximately 15 min). Patients in group (B) are required to turn up for CET group therapy every other week for 8 weeks (four sessions of 120 min each, with a maximum of eight patients in each group). Patients in group (C) will receive 1 individual follow-up session 8 weeks after discharge from CBT.

### CET aftercare

CET is most commonly used in combination with various urge-specific coping strategies (USCS) because this has been shown to provide better treatment outcomes [13, 14]. In this study, we will apply the treatment manual for CET with USCS recommended by Monti and colleagues [14], which emphasizes the importance of patients being confronted with alcohol in order for cue reactivity to diminish. During each CET session, the patient is introduced/reintroduced^1^ to effective USCS and afterwards required to practise the strategies learned whilst being exposed in vivo to alcohol. The recommended strategies are as follows: 1) waiting it out; 2) thinking about the negative consequences of drinking; 3) thinking about the positive consequences of sobriety, and 4) alternative food and beverage intake. In a recent study, negative consequences of drinking and positive consequences of sobriety were identified as the most effective USCS related to treatment outcome among 13 such strategies, whereas alternative food and beverage intake was surprisingly not even significantly related [15]. However, other studies have reported good effects for all of them [10, 16, 17], and further research would be needed to exclude any of them from the CET treatment program. Both experimental groups, (A) and (B), will receive manual-based CET since CET is suitable for individual as well as group therapy.

#### CET aftercare via a smartphone application

**Fig. 2:**
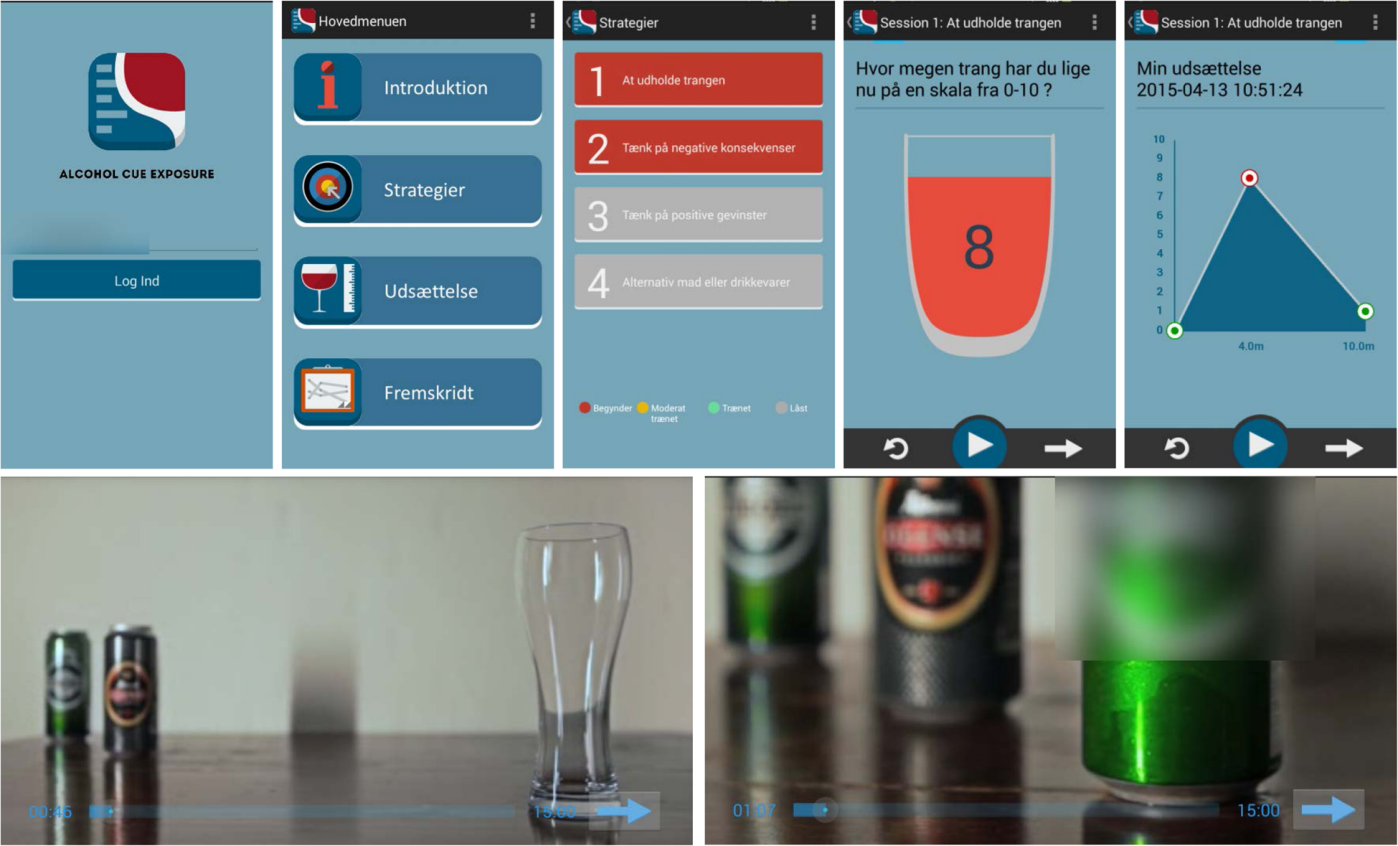
Smartphone app illustration

Based on the Monti et al. [14] manual, CET is provided in some treatment programmes in Danish alcohol clinics in both outpatient and inpatient treatment settings. Due to the structure of the treatment and clinical experience with the method, we have been able to transform the method into a smartphone application (Fig. 2). The application is individually adaptive in terms of the contents of USCS as well as the alcohol exposure material.

The exposure to alcohol is simulated by watching one of ten alcohol videos on the smartphone (beer, red or white wine, mixed alcoholic drinks, hard liquor etc.…), which allows the patient to select his/her preferred beverage as exposure material. The alcohol exposure videos imitate sessions with a therapist, and the alcohol presented in the videos becomes increasingly appetitive during the exposure session, in order to induce cue controlled cravings. The application contains a direct number to a CET therapist in case of uncontrollable cravings, and the app is only accessible during the opening hours of the alcohol outpatient clinic (Monday to Friday from 9 to 18). Patients are able to practise exposure once a day, four times a week, and they are sent a reminder by text message from the software 4 times a week. Exposure activities as well as effectiveness will be measured by algorithms of cravings during exposure, coupled to a data-monitoring system.

The application has the advantage of allowing patients to practise exposure at home and in a variety of real-life situations. Compared to the CET treatment delivered in alcohol clinics, this approach may increase the likelihood that the extinction learning will generalize to various other contexts outside the usual treatment setting.

#### Aftercare as usual

The usual aftercare consists of a single follow-up session, during which the patient is asked how he/she is doing and, if needed, offered a brush-up of the coping strategies.

### Measures

Demographic data are recorded for all patients when entering alcohol treatment. Assessment for harmful use and dependence on alcohol by means of the ICD-10 Diagnostic Criteria for Research (DCR-10) and the Addiction Severity Index (ASI) also forms part of the routine clinical assessment and is carried out for all patients when entering primary treatment, and at conclusion. Typically, more than 90 % of the patients suffer from alcohol dependence at treatment entry (Data from Alkoholbehandlingen i Odense, 2011).

Patients suffering from severe dementia or acute psychosis are not offered treatment at the outpatient clinic but referred elsewhere. At the conclusion of the primary treatment and before randomization, patients fulfilling the exclusion/inclusion criteria will be further assessed by means of the primary and secondary treatment outcomes in order to be able to measure the effect of the CET aftercare treatment. The primary and secondary outcome measures are presented in Table 1: Measurements.

**Table 1:** Measures

Domain	Measure	Content	Reference	Time-point
Primary outcome				
Alcohol consumption	Timeline Followback (TLFB)	TLFB is used to identify alcohol-free days as well as number of drinks per day. Patients indicate their daily standard drink intake before administration.	[26]	Baseline and follow-up (after 8 and 26 weeks)
Secondary outcomes				
Addiction severity	Addiction Severity Index (ASI)	ASI assesses characteristics and problems in various domains of life; e.g. alcohol and drug use module – and also physical and mental health, employment, legal problems and social functioning.	[27]	Baseline and follow-up (after 8 and 26 weeks)
Craving	Desires for Alcohol Questionnaire (DAQ)	DAQ measures three dimensions of current craving: desire and intention to use alcohol; negative reinforcement of alcohol use, and control of alcohol use.	[28]	Baseline and follow-up (after 8 and 26 weeks)
	Obsessive Compulsive Drinking Scale (OCDS)	The OCDS is designed to reflect obsessive patterns and compulsivity related to craving and drinking behaviour.	[29]	Baseline and follow-up (after 8 and 26 weeks)
	The Visual Analogue Scale for Craving (VAS)	VAS measures on the subjective experience of the intensity of current craving for alcohol.	[30]	Baseline and follow-up (after 8 and 26 weeks)
Coping skills and self-efficacy	The Urge-Specific Strategies Questionnaire (USS)	USS is designed to assess the patient’s use of coping skills when trying to stop themselves from drinking after experiencing an urge.	[14]	Baseline and follow-up (after 8 and 26 weeks)
	Alcohol Abstinence Self-Efficacy Scale (AASE)	The AASE assesses self-efficacy and evaluates an individual’s efficacy (e.g. confidence in their ability) to abstain from drinking.	[31]	Baseline and follow-up (after 8 and 26 weeks)

Data will be collected at three different points in time: T_0,_ the baseline (which in this case is after conclusion of treatment, but before entering aftercare); T_1,_ after having completed aftercare treatment, 8 weeks after the baseline assessment, and T_2,_ 26 weeks after baseline. T_2_ is the primary time endpoint.

In addition to the measurements 8 and 26 weeks after CET aftercare or control group, patients will be followed up 1 year after concluding aftercare treatment through the following population registers: The Danish National Patient Register [18], 2011), The Danish National Health Service Register [19], The Danish National Prescription Registry [20] and The Danish Psychiatric Central Research Register [21]. The registers provide data about contact to the healthcare system as well as reason for contact and prescriptions during the period, which will enable us to detect relapses.

The cost-effectiveness of CET by smartphone intervention will be compared by healthcare expenditure per primary outcome unit in group (A) versus groups (B) and (C). Healthcare expenditure is measured according to the international Classification of Health Accounts [22], and almost all components can be extracted from the above-mentioned population registers.

### Statistical analyses

The primary outcome measure is alcohol consumption, comprising a number of drinking variables such as abstinence and controlled drinking. In Denmark, sensible drinking, defined by the Danish Board of Health as ≤ 14 drinks per week for women and ≤ 21 drinks^2^ per week for men, is the most common measure of abstinence or controlled drinking. Therefore we have selected sensible drinking as our primary outcome (26 weeks after baseline assessment). Because no similar study has been conducted using this variable, the power calculation is estimated from quality assurance data and research data from an alcohol outpatient clinic.

Currently, 26 weeks after starting treatment in the current treatment regime, 65 % of the patients have sensible drinking habits (Data from Alkoholbehandlingen i Odense, 2011). In order to detect an effect by comparing the three groups, a sample of 100 patients in each group is needed to have 90 % power of detecting a difference corresponding to an improvement of 18 percentage points using a 5 % level of statistical significance.

The primary and secondary outcome data will be analysed by repeated measures and mixed models in order to detect differences within and between the three groups. The significance level is set at 5 %, and either one-sided or two-sided analyses will be applied depending on the hypothesis being modelled (i.e. a priori or post-hoc). Effect sizes will be reported in accordance with the statistical modelling. For each outcome measure, two analyses will be carried out: 1) multiple imputation analyses will be carried out for all patients, irrespective of whether they have completed the interventions or were re-interviewed. 2). Completer (on-treatment) analyses will be carried out for patients who have completed the respective interventions.

### Ethics

Usually, the Aftercare as Usual treatment for AUD today involves either no aftercare at all or only a single booster session 8 weeks after discharge. Therefore, we find no ethical problems in studying the effect of adding aftercare to treatment. The only critical ethical question in this study is whether the use of the smartphone application as aftercare may place the patient at risk of relapse, instead of preventing it. This concern relates to the fact that the application involves modelling in vivo exposure through the alcohol videos. However, in Danish culture, all patients will be exposed to alcohol, both during and after discharge of treatment, and they will be unable to avoid this since large-scale alcohol advertisements are on display in the public space, in magazines and on television. Also, alcohol is available, highly visible and easy to buy around the clock, in all supermarkets, delicatessens, kiosks and gas stations. We therefore consider the exposure to alcohol videos in the experimental groups to be no more risky for the patients than their exposure in everyday life. On the contrary, in the experimental groups, the patients may face alcohol and high-risk situations in a better prepared and more conscious way. Furthermore, the application is only available during the opening hours of the alcohol outpatient clinic and contains a direct number to a CET therapist in case of uncontrollable cravings, whereby all precautions will be taken to intervene if the patient relapses.

The study protocol has been approved by the Regional Scientific Ethical Committee for Southern Denmark and the Danish Data Protection Agency. All procedures in the study are in accordance with the second Declaration of Helsinki.

## Discussion

The specific aim of this study is to investigate whether manual-based CET as aftercare in extension of primary treatment with CBT has an effect, measured by a number of parameters: alcohol consumption, cravings, coping skills/self-efficacy and cost-effectiveness.

The extent to which CET is an effective intervention for substance use disorders (SUD) in general has been heavily debated during the last decade [23, 24]. However, when specifically targeting AUD, Randomized Controlled Trials (RCT) have reported significant improvement and medium effect for alcohol consumption outcomes when using CET compared to treatment as usual [17] and to relaxation/meditation treatments [10, 16, 25]. Furthermore, the majority of these studies indicate that the CET approach may be particularly effective in the long term, which makes it appropriate in aftercare relapse prevention. Only one of these studies delivered CET as aftercare following primary alcohol treatment, but reported significantly fewer relapse days as well as heavy drinking days at 6- and 12-month follow-up [10], also indicating that CET as aftercare may be a suitable strategy.

CBT often comprises CET when targeting psychopathology, for instance anxiety disorders, but there appears to be a segregation of these treatments methods in the empirical literature when it comes to SUD/AUD. When the methods are segregated in AUD literature, the difference emphasized between CET and CBT is the in vivo exposure element in CET. However, it could be argued that there seems to be some confusion; CET is indeed most often integrated within the CBT framework as one of the behavioural methods when treating other types of psychopathology, and it may be difficult to point out the difference when it comes to AUD. This is particularly the case when the CET method is combined with the use of coping strategies, as in the majority of prior studies. In continuation of the prevailing segregation, a number of RCT studies comparing the effect of CBT with CET have been performed. Out of a total of five RCT studies, four reported the effect of CET on alcohol consumption outcomes as being equal to CBT [4, 5, 7, 8], whilst one study, albeit with the smallest sample size, reported the effect of CET on alcohol consumption outcomes as being significantly superior to CBT [6]. Thus, when comparing CET to CBT, the outcomes appear less promising compared to treatment as usual and relaxation/meditation treatments, which may be at least partly due to their similarity. Also, the majority of the CBT studies are based on participants with only minor alcohol problems, the goal being to moderate their alcohol consumption and not total abstinence. In fact, this is in contrast to the studies comparing CET with treatment as usual or relaxation techniques targeting AUD. The effects of CET and CBT comparison studies may therefore be problematic to compare with the effects of interventions designed to target severe AUD/alcohol dependence. For instance, Loeber and co-authors [8] reported no overall difference between groups, but provided preliminary evidence suggesting that CET may be superior to CBT among inpatients with more pronounced AUD. Actually, this was the only one of the reviewed CBT studies that investigated on alcohol dependent patients.

Even though there is heterogeneity in CET approaches and patient samples, it is puzzling that CETs do not produce significantly better alcohol intake outcomes than CBT in more studies. However, similar to all but one study comparing CET with treatment as usual and relaxation/meditation treatments, these CBT studies were not part of an aftercare programme. The in vivo exposure may be particularly suitable as an aftercare treatment for patients with AUD or potentially the most severe AUD cases. CET may allow patients to practise exposure and gain control over alcohol cue reactivity and associated high-risk situations in an inter-mediating therapeutic context before the patients are inevitably confronted with them in real life. Thus, it might be expected that the transition from treatment to daily life is less overwhelming for the patients, and that CET may help prevent relapse in the long term. Moreover, the CET application has the advantage of allowing the patient to practise when it is convenient, at home as well as in a variety of situations in real life, which may increase compliance. Also, compared to the CET treatment delivered in alcohol clinics, this approach may facilitate that extinction learning is generalized to various other contexts outside the usual treatment setting. Potentially, this could further decrease the challenges associated with the transition from treatment to everyday life.

In recent years, primary AUD treatment has been changing, at least in Denmark, due to the increasing socio-economic burden of mental healthcare. The duration of treatment is being shortened, and there is a tendency towards moving away from individual to group sessions. Furthermore, we see a huge interest in integrating healthcare technology in treatment. Since almost everyone has a mobile phone these days, it makes sense to prolong individual treatment by means of this technology, if possible. Also, if the CET application proves effective, it can be introduced as a non-stigmatizing treatment tool, which can be easily integrated into patients’ everyday life.

By targeting AUD, this large-scale RCT study will advance our understanding on several aspects, the most important being the effectiveness of CET as aftercare, the effectiveness of CET aftercare within a CBT framework, and the effectiveness of CET as a smartphone application in relation to costs. If the study verifies the hypothesis that CET increases the effect of CBT, we will recommend reintegrating this approach as aftercare within a CBT framework. Moreover, if CET by means of smartphone application proves recommendable, it can be a helpful non-stigmatizing tool to increase the availability of evidence-based psychological aftercare treatment as well as help decrease the socio-economic burden on the healthcare system in the future.

### Availability of data

All data entry and databases are based on REDCap (Research Electronic Data Capture), and the Odense Patient data Explorative Network (OPEN) are managing the databases of the present study.

## Footnotes

1 Many of the coping strategies have already been introduced during CBT.

2 1 standard drink is equivalent to 12 grams of ethanol.
